# Using the Consolidated Framework for Implementation Research (CFIR) to produce actionable findings: a rapid-cycle evaluation approach to improving implementation

**DOI:** 10.1186/s13012-017-0550-7

**Published:** 2017-02-10

**Authors:** Rosalind E. Keith, Jesse C. Crosson, Ann S. O’Malley, DeAnn Cromp, Erin Fries Taylor

**Affiliations:** 1 Mathematica Policy Research, PO Box 2393, Princeton, NJ 08543 USA; 2 Mathematica Policy Research, 1100 1st Street, NE, 12th Floor, Washington, DC 20002 USA; 3 Group Health Research Institute, 1730 Minor Ave. Ste. 1600, Seattle, WA 98101 USA

**Keywords:** Implementation framework, Barriers and facilitators, Qualitative methods, Practice transformation, Primary care redesign, Rapid-cycle evaluation, Actionable findings

## Abstract

**Background:**

Much research does not address the practical needs of stakeholders responsible for introducing health care delivery interventions into organizations working to achieve better outcomes. In this article, we present an approach to using the Consolidated Framework for Implementation Research (CFIR) to guide systematic research that supports rapid-cycle evaluation of the implementation of health care delivery interventions and produces actionable evaluation findings intended to improve implementation in a timely manner.

**Methods:**

To present our approach, we describe a formative cross-case qualitative investigation of 21 primary care practices participating in the Comprehensive Primary Care (CPC) initiative, a multi-payer supported primary care practice transformation intervention led by the Centers for Medicare and Medicaid Services. Qualitative data include observational field notes and semi-structured interviews with primary care practice leadership, clinicians, and administrative and medical support staff. We use intervention-specific codes, and CFIR constructs to reduce and organize the data to support cross-case analysis of patterns of barriers and facilitators relating to different CPC components.

**Results:**

Using the CFIR to guide data collection, coding, analysis, and reporting of findings supported a systematic, comprehensive, and timely understanding of barriers and facilitators to practice transformation. Our approach to using the CFIR produced actionable findings for improving implementation effectiveness during this initiative and for identifying improvements to implementation strategies for future practice transformation efforts.

**Conclusions:**

The CFIR is a useful tool for guiding rapid-cycle evaluation of the implementation of practice transformation initiatives. Using the approach described here, we systematically identified where adjustments and refinements to the intervention could be made in the second year of the 4-year intervention. We think the approach we describe has broad application and encourage others to use the CFIR, along with intervention-specific codes, to guide the efficient and rigorous analysis of rich qualitative data.

**Trial registration:**

NCT02318108

**Electronic supplementary material:**

The online version of this article (doi:10.1186/s13012-017-0550-7) contains supplementary material, which is available to authorized users.

## Background

Numerous health care delivery interventions are being implemented across the USA with the aim of achieving better health outcomes for patients at lower costs. These health care delivery interventions are as complex as the issues in health care that they are designed to improve. Further, the contexts in which they are implemented are increasingly complex, involving interdependent interactions within and across delivery organizations. Understanding how primary care practices are faring when implementing these multifaceted interventions requires systematic research that yields information on the factors that emerge to influence implementation [1–3]. However, much research does not address the practical needs of the stakeholders responsible for introducing these interventions into the health care delivery organizations working to achieve better outcomes [4, 5]; these stakeholders can include payers and providers, and those helping providers alter care delivery. Rapid-cycle evaluation provides stakeholders with timely assessment of intervention effectiveness and ongoing feedback to support continuous improvement of an intervention during the implementation period to maximize its effectiveness [6].

### The Consolidated Framework for Implementation Research

The Consolidated Framework for Implementation Research (CFIR) is a conceptual framework that was developed to guide systematic assessment of multilevel implementation contexts to identify factors that might influence intervention implementation and effectiveness [7]. A conceptual framework is a system of concepts, assumptions, expectations, beliefs, and theories that “explains either graphically or in narrative form, the main things to be studied—the key factors, constructs or variables” that influence a phenomenon of interest [8]. Conceptual frameworks increase the efficiency of research and the generalizability and interpretability of research findings. By prespecifying factors demonstrated in prior research to influence the phenomenon of interest, in this case implementation of an intervention to improve health care delivery, conceptual frameworks increase the relevance of the research findings for informing implementation practice. To develop the CFIR, Damschroder et al. [7] reviewed many published implementation theories and reports of empirical studies to identify factors associated with effective implementation. They considered a spectrum of construct terminology and definitions, from them compiled an overarching framework. The 39 CFIR constructs reflect the evidence base of factors most likely to influence implementation of interventions. The CFIR is well suited to guide rapid-cycle evaluation of the implementation of complex health care delivery interventions, because it provides a comprehensive framework to systematically identify factors that may emerge in various, multi-level contexts to influence implementation. If used to evaluate the initial stages of implementation, the CFIR can help to produce findings to inform stakeholders on improvements to the intervention and its implementation. Since its publication in 2009, the CFIR has been cited in more than 300 published articles [9]. Damschroder and Lowery describe an approach to using the CFIR to explain variation in the implementation of an intervention and compare the influence of CFIR constructs on implementation across studies [10]. However, we are not aware of any other journal articles that describe how to use the CFIR to systematically assess barriers and facilitators to implementation for rapid-cycle evaluation in which actionable findings are shared with stakeholders during implementation.

The CFIR is composed of five major domains, each of which may affect an intervention’s implementation [7]:

1. Intervention characteristics, which are the features of an intervention that might influence implementation. Eight constructs are included in intervention characteristics (e.g., stakeholders’ perceptions about the relative advantage of implementing the intervention, complexity).
2. Inner setting, which includes features of the implementing organization that might influence implementation. Twelve constructs are included in inner setting (e.g., implementation climate, leadership engagement).
3. Outer setting, which includes the features of the external context or environment that might influence implementation. Four constructs are included in outer setting (e.g., external policy and incentives).
4. Characteristics of individuals involved in implementation that might influence implementation. Five constructs are related to characteristics of individuals (e.g., knowledge and beliefs about the intervention).
5. Implementation process, which includes strategies or tactics that might influence implementation. Eight constructs are related to implementation process (e.g., engaging appropriate individuals in the implementation and use of the intervention, reflecting, and evaluating).

The CFIR is intended to be flexible in application so that researchers can tailor the framework to the specific intervention design, factors, and context being studied.

In this article, we describe how we used CFIR to guide data collection, analysis, and reporting of actionable findings related to the implementation of the Comprehensive Primary Care (CPC) initiative. The CPC initiative, launched by the Centers for Medicare and Medicaid Services (CMS) in 2012, is a 4-year multipayer initiative designed to strengthen primary care to improve health, lower costs, and patient and provider experience. CMS collaborated with 39 commercial and state health insurance plans in seven regions across the USA to provide to the 497 participating primary care practices’ financial support in the form of monthly care management fees (in addition to regular fee-for-service payments) and opportunities to share in any savings. In addition to this financial support, CPC provided regular data feedback and learning support focused on guiding required improvements in the delivery of five primary care functional areas by participating practices: access to and continuity of care; planned care for chronic conditions and preventive care; risk-stratified care management; engagement of patients and their caregivers; and coordination of care with patients’ other care providers [11]. CMS defined nine annual milestones that participating practices were required to meet in the process of implementing CPC, thereby changing care delivery and building capacity across these five functional areas, referred to in this article as program components. In Table 1, we provide a brief overview of the five CPC program components and an illustrative supporting milestone for each.

**Table 1:** Comprehensive Primary Care components and illustrative supporting milestones for 2013

Primary care component	Definition and supporting milestone activities
1. Access and continuity	The primary care practice ensures that the patient has 24/7 access to speak with a practitioner or nurse who has access to the practice’s EHR system and ensures continuity between the patient and the PCP and care team. Milestone: Practice defines the infrastructure (both technology and staffing) that supports 24/7 real-time access to practice’s EHR system.
2. Planned care for chronic conditions and preventive care	The primary care practice proactively assesses patients to determine care needs and provide appropriate and timely chronic and preventive care, including medication management and review. Milestone: A care team member develops a personalized plan of care for high-risk patients and uses team-based approaches to meet patient needs efficiently.
3. Risk-stratified care management	The primary care practice delivers and manages care for patients with complex care needs (e.g., chronic illness and/or multiple comorbidities). The primary care practice empanels and risk stratifies its practice population and provides care management services to high-risk patients. Milestone: Practice develops a risk stratification process and reports on the status of empanelment, data on the number of patients within each risk stratum, and information about care management processes, such as forming care teams or identifying and recruiting high-risk patients to receive care management services.
4. Patient and caregiver engagement	Primary care practice engages patients and their families in active participation in patient care and in guiding improvement in the system of care. Milestones: Practice conducts an assessment of patient- and family-centered care and then engages in improvement activities informed by either conducting a practice-based survey or forming a patient and family advisory council.
5. Care coordination across the medical neighborhood	Primary care practice is the first point of contact for many patients and takes the lead in coordinating care as the center of patients’ experiences with medical care. Practice works closely with patients’ other health care providers, coordinating and managing care transitions, referrals, and information exchange. Milestone: Practice identifies one area of care coordination (post-hospital discharge visit, emergency department follow-up phone call or visit, or referral tracking for specialist visits) for improvement and tracking.

*EHR* electronic health record, *PCP* primary care provider

We demonstrate how our approach facilitated understanding of practice transformation efforts to support improvements in implementation of the CPC initiative. We provide examples to illustrate the analytic methods we used to develop our findings, and we reflect on the utility of our approach for producing actionable findings for decision makers and program implementers. Details about the initiative and the results of the evaluation are presented elsewhere [11–14].

## Methods

We conducted a formative cross-case qualitative investigation of the implementation of the CPC initiative, a primary care practice transformation initiative. We present the steps in our approach to using the CFIR to guide the collection of rich qualitative data, rapid-cycle data analysis, and the reporting of actionable findings about contextual and intervention factors affecting implementation and intervention outcomes during 2013, the 2nd year of our 5-year evaluation.

One of the objectives of the evaluation was to understand how participating practices were experiencing the implementation of changes in the five primary care functional areas. The specific research objectives around practice transformation were to (1) describe the changes being made by practices to implement the five core program components; (2) describe tactics used by practices to make those changes; and (3) identify barriers and facilitators’ practices faced when implementing the changes. Our research team was comprised of a medical anthropologist, two primary care physician researchers, two social scientists with expertise in primary care transformation and implementation science, and several research analysts. Three of the authors (REK, JCC, DC) collected, coded, and analyzed the data. Three of the authors (REK, JCC, ASO) reported findings.

### Data collection

To select practices, we stratified practices in each region by size (small, medium, and large practices). Within each category in each region, we randomly selected two practices, which we designated as primary, and another practice that we designated as an alternate. We used these 14 primary and 7 alternate practices to roughly approximate the distribution we had for all CPC practices on other characteristics such as ownership, rural or urban location, and previous medical home recognition status. This process ensured that we included roughly equal numbers of small, medium, and large practices. The selected practices were broadly similar to the overall group of CPC participating practices in terms of ownership, rural or urban location, and previous medical home recognition status. We then visited each of the 21 selected practices between June and October 2013 for 1 or 2 days (depending on practice size). During these visits, we used semi-structured interview guides to conduct in-depth interviews with multiple respondents, including lead clinicians, practice managers, other clinicians, and administrative and medical support staff involved in CPC-related practice functions. In small practices, we typically interviewed all available staff; in larger practices, we typically interviewed five to seven respondents to ensure that we had a variety of perspectives on CPC implementation. Interviews were 30 to 90 min long. All interviews were audio-recorded and transcribed verbatim.

We designed our interview guide to ensure that we collected data related to our research questions. Our semi-structured interview guide prompted respondents to discuss their practice context as it related to their experiences with implementing individual CPC components, and we probed respondents regarding the challenges and facilitators they experienced when implementing each component. We did not ask questions about specific CFIR constructs; rather, we asked respondents questions about their experiences with each of the five CPC core components. We asked respondents about (1) how each component was being operationalized in their practice; (2) how practice functions and workflows supported (or did not support) each component; (3) what had been challenging with operationalizing each component; (4) what had been helpful with operationalizing each component; and (5) how patients were reacting to each component. We provide an excerpt from the semi-structured interview guide in Additional file 1.

In addition to the in-depth interviews, we developed a checklist to guide observation of the practice context and CPC-related workflows and informal interviews with administrative and medical support staff. Our checklist prompted us to note observations of the practice context by CFIR domain, including (1) the inner setting of the practice context, (2) practice members’ perceptions of CPC milestones, (3) the practice’s process for implementing CPC milestones, and to some extent (4) the practice’s outer setting. We shadowed additional clinical and administrative support staff and conducted informal interviews to clarify things that were discussed during the semi-structured interviews. The observations and informal interviews provided perspective on the extent to which CPC’s goals and changes in workflows to meet those goals were understood and supported by staff throughout the practice. We obtained as complete of a picture as possible of what CPC implementation looked like in the practice. We documented field notes within 24 h after each site visit. Field notes from these observations and informal interviews were included in our data analysis. We provide an excerpt from the observation checklist in Additional file 2.

We pilot tested the interview guide and observation checklist and made refinements based on pilot respondent feedback and research team members’ perceptions of the usefulness of the data collection instruments for eliciting information we intended to capture. We then used these refined data collection instruments for the interviews and observations reported on here.

### Coding

We used a template analysis approach to code and organize our data for analysis. The template analysis approach involves using a coding template (or codebook) to balance the structure involved in using a framework to analyze data with the flexibility necessary to adapt the codebook to the study context [15]. We developed two codebooks before coding the data. In one codebook, we defined operational codes for each of the five CPC program components. These operational codes were descriptive in that their definitions were based on implementation guidelines developed by CMS for practices participating in the initiative. Defining these operational codes enabled us to focus on the distinct CPC components (defined in Table 1) and use matrix data displays during subsequent analysis of coded data (which we explain below) [8, 16]. Examples of two of the five operational codes are included in Table 2. In the second codebook, we initially included all 39 CFIR constructs and their definitions as codes to capture contextual factors that might influence the implementation of CPC components. These CFIR codes were analytical in that they required the coder to interpret the data and then apply the CFIR code that reflected a potential barrier or facilitator being described, which was the main theoretical driver of our study [8, 16].

**Table 2:** Example operational codes

CPC program component	Definition and coding rules
Risk-stratified care management	A primary care practice’s management of care for patients with complex care needs (e.g., chronic illness or multiple comorbidities). Under this component, practices are expected to deliver care management services for patients with high needs or complex needs (i.e., high-risk patients). Code discussion of: • Assigning a risk status to patients on the panel and documenting each patient’s risk status in the electronic health record • Tracking the percentage of patients assigned a risk status and the proportion of the panel in each risk category • Generating lists of patients by risk category • Reporting on which high-risk patients have received care management services • Forming care teams • Providing care management services • Identifying and recruiting high-risk patients to receive care management services • Developing or using a personalized plan of care for each patient • Managing or reconciling medications • Delineating roles of staff who provide care management (e.g., a care coordinator or care manager)
Care coordination (across the medical neighborhood)	A primary care practice’s coordination of patient care with other health care providers. This includes ensuring that patient information necessary for providing care is available across the medical neighborhood (i.e., to other providers who care for the patient). This also includes following up with patients who have been discharged from a tertiary care facility. Code discussion of: • Selecting a priority area/care interface/transition (e.g., hospital or emergency department (ED)) for care coordination • Following up with patients after hospital or ED discharge • Establishing referral compacts and information-sharing arrangements with other providers, including specialists and diagnostic testing facilities

During initial coding of six transcripts and following the template analysis approach described above, we adapted the CFIR codes to fit the context of CPC and our study in three ways. First, we removed a small number of CFIR codes that were not reflected in the transcripts. For example, we removed codes for “trialability” and “individual stage of change” from the codebook. Second, we made minor modifications to CFIR code definitions to use language found in our data, but did not change the meaning of any CFIR construct. For example, we modified the definition of “available resources” to fit the context of CPC implementation by creating two additional “available resources” codes: “staff resources” and “health information technology resources.” Third, we added examples in the codebook to ensure consistent application of codes over time and across coders. An example of a data segment we added to the codebook is this segment illustrating the use of the code “patient needs and resources”:

[Interviewer: “What are the challenges with providing care management?”]

Respondent: “We’re working on it slowly, kind of. But one of the things is patient transportation. So many of our patients are elderly, live alone, and have really no … social outlet, network, or anything like that. So we’ve been working on ways of getting them to appointments … and you get a call a day from them, with nothing you can really do without seeing them, but they can’t come in, because they can’t get here.”

We then used the adapted codebooks to code the remaining data.

To reduce the data for analysis, coders were judicious in applying the fewest codes possible in interpretation of the meaning of each data segment [17]. When coding the data, coders made three decisions for each data segment. (Data segments typically included an interview question and response or a single paragraph of a field note.) First, the coder determined which of the five CPC components was being discussed and assigned the appropriate operational code (e.g., care coordination). Second, the coder identified which one of the five CFIR domains reflected the principal implementation theme in the data (e.g., intervention characteristic). Third, the coder determined which CFIR code within that identified domain was reflected in the data segment and assigned the appropriate contextual code (e.g., relative advantage). In this third step, coders applied codes to capture the principal implementation theme in the data segment, by applying only one CFIR code per CFIR domain. While the use of one CFIR domain per data segment was our general rule, coders made exceptions if two CFIR domains were equally reflected in the data segment; specifically, coders selected both domains and applied a CFIR code for each, but did not apply more than one CFIR code per domain. This approach helped us to increase coding consistency and avoid overapplying codes, by focusing our interpretation on the most relevant CFIR constructs found in the data. We found that limiting the number of codes we applied to each data segment effectively reduced the large amount of data we had to analyze by ensuring that the same data segment was not being analyzed multiple times within different CFIR constructs. In addition, we found that using this hierarchical decision process, and in most cases narrowing in on the most relevant CFIR domain, made using the CFIR codes more manageable, because it helped to reduce the cognitive burden of having to recall all of the CFIR codes from all five domains, while coding.

A concern we had about limiting the number of CFIR codes we applied to each data segment was losing data that potentially reflected more than one CFIR construct. We explain below, under the “Data analysis” section, how we used the operational codes to organize the coded data by program component for analysis. This ensured a comprehensive analysis of all data segments relevant to each program component, while avoiding having to review the same data segment multiple times.

In the initial stages of coding, a team of four researchers coded data together. As part of this process, the coders refined code definitions, developed coding rules, and achieved agreement on the application of codes to the data. After this process, the remaining transcripts were divided among three coders who, after each coding five different interviews, coded the same sixth interview independently and met to discuss and resolve coding discrepancies to ensure ongoing consistency in the application of the codes. The coders continued this process of each coding five different interviews and then coding and discussing the same sixth interview, until all data were coded. All data were coded in ATLAS.ti [18].

### Data analysis

To analyze the coded data, we first generated code reports from ATLAS.ti for each practice that included all the data segments coded for each combination of program component and CFIR construct. Within each code report, data segments were organized by CFIR domain and construct. For example, the code report for “care coordination” included all coded data focused on how the practice made changes to implement care coordination and what barriers and facilitators (identified by CFIR construct) that the practice experienced in making those changes, grouped by each of the CFIR domains (e.g., intervention characteristic). We then developed analytic summaries for each combination of program component (e.g., care coordination) and CFIR construct (e.g., relative advantage, complexity) for each of the 21 practices and determined whether the construct exerted a negative, positive, or neutral influence on implementation. For example, we considered if the complexity described in the data segment as a feature of CPC reflected a positive or negative influence on the implementation of care coordination. The guidance we developed and used to assess the direction of the influence on implementation is provided in Additional file 3.

We then populated analytic matrices with this information for cross-case analysis of patterns of barriers and facilitators relating to each of the program components (see Table 3 for an example analytic matrix display for the care coordination component) [19]. Our analytic matrices facilitated simultaneous viewing of a large volume of data so we could make between-practice comparisons and identify similarities, differences, and trends in how practices experienced implementation.

**Table 3:** Example analytic matrix for the care coordination component

Practice	Intervention characteristics	
	Relative advantage	Complexity
A	(+) Medical director describes the benefit of the nurse’s calling patients upon hospital discharge and talking with them about their medications before they come in for a visit. Prior to the Comprehensive Primary Care initiative, he would spend the whole patient visit trying to figure out the medications of a recently discharged patient, “instead of actually taking care of them.”	(+/−) Nurse reports that the specialists in the community are generally good about sending patient information to the practice after a visit. The practice does have to track down some information, which she notes is one of the harder things to do, but at the same time, the practice is getting better at referral tracking. “Because it’s out of your control. You’re dependent on somebody else. You know, to get that. But as we get better and better at our referral tracking, that will flow a little bit easier, too.”
B	(+) Nurse reports that having staff to follow up with high-risk patients after a hospitalization improves the care that the practice can provide for these patients going forward.	(−) Practice manager reports that not having an electronic interface with other care settings to exchange patient information means that the practice had to develop a process for collecting this information manually, scanning the records into the electronic health record, and then making sure that key information is manually entered into discrete fields in the electronic health record for appropriate tracking.

+, −, and +/− signs at the start of each data segment example indicate whether the construct exerted a positive, negative, or neutral influence on implementation

In Table 3, the analytic summaries for relative advantage reflect a facilitator that emerged from the data about respondents’ perceptions regarding the advantages of implementing care coordination. The analytic summaries for complexity reflect a barrier that emerged from the data about the challenges with implementing care coordination.

### Reporting the findings

Drawing on the analytic matrices for each program component and CFIR domain combination, we described patterns of barriers and facilitators to implementation as they emerged across the 21 practices. Our reporting of findings conveyed the richness of the qualitative information and preserved the complexity of these patterns while maximizing learning across practices. We did not use CFIR terminology to report our findings but rather we framed key findings in language familiar to our audience.

In addition to our narrative report of these findings, we developed a summary table of barriers and facilitators to implementation as they emerged across the 21 practices, organized by CFIR domain, across each of the five CPC components (Table 4) [12]. This table identifies barriers or facilitators that were common across the program components, as well as those that were unique to each component. Visualizing barriers and facilitators in this manner may be helpful for identifying key areas where additional support could be important for implementation success.

**Table 4:** Facilitators and barriers to implementation across the five CPC components, as commonly reported or observed in deep-dive practice interviews and visits conducted in 2013

CFIR domain	CPC component				
	Access and continuity	Planned care for chronic conditions and population health	Risk-stratified care management	Patient and caregiver engagement	Coordination of care
Characteristics of the CPC initiative					
Facilitators					
Adequate resources for new capacities (both financial and time)	✓	✓	✓	✓	✓
Compatibility with care improvement objectives			✓		
Barriers					
Insufficient resources for new capacities (tools, financial, time)			x	x	
Complex or unclear requirements			x	x	
External environment and context					
Facilitators					
Effective local electronic HIE		✓	✓		✓
HIT “meaningful use” incentives	✓				
Regional history of patient-centered medical home programs	✓	✓	✓	✓	✓
Barriers					
Lack of direct electronic access to health information from other care settings		x	x		x
Delays in access to patient survey results				x	
Gaps in electronic information available through HIE		x	x		x
Complexity of needs in patient population			x		
Internal context and setting of the practice					
Facilitators					
Prior experience with quality improvement efforts	✓	✓	✓	✓	✓
Organizational commitment to population health approaches to care		✓	✓		
Independent practices could make rapid change	✓	✓	✓	✓	✓
System-affiliated practices had support for management, HIT, quality improvement		✓	✓		✓
Integration of new work with existing work processes			✓		
EHR technology integrated with disease registries and patient reminder systems		✓	✓		
Prior use of shared decision-making tools		✓	✓		
Existing staff trained in patient self-management approaches				✓	
Barriers					
Organizational commitment to traditional office visit-driven model of care		x	x		
Independent practices lacked support for management, HIT, and quality improvement			x		
System-affiliated practices had limited local authority to make change	x	x	x	x	x
Lack of a practice-level quality improvement infrastructure	x	x	x	x	x
Lack of population management systems and sufficient care management staffing			x		
Lack of knowledge of available shared decision-making tools		x		x	
Preventive health and chronic illness-related data entered into EHRs as unstructured data		x	x		
EHRs had to be modified to integrate new work			x	x	
Characteristics and attitudes of practice staff and clinicians					
Facilitators					
Shared staff and clinician commitment to population health approaches to care		✓	✓		
Barriers					
Clinician skepticism regarding the value of CPC requirements			x	x	
Shared staff and clinician commitment to office visit-driven model of care		x			
CPC implementation process within the practice					
Facilitators					
Use of established quality improvement processes	✓	✓	✓	✓	✓
Use of pilot testing before making practice-wide changes	✓	✓	✓	✓	✓
Tailored assistance from regional learning faculty				✓	
Standardization of implementation processes across system-affiliated practices	✓	✓	✓	✓	✓
Dedicated CPC implementation meetings	✓	✓	✓	✓	✓
Barriers					
Implementation limited to some (not all) clinicians or care teams, creating multiple workflows for the same processes		x	x	x	x
Knowledge of CPC requirements unevenly shared across practice members		x	x	x	x

Source: [12]. For each CPC component where they apply, facilitators are indicated with a checkmark and barriers are indicated with an x. *CPC* Comprehensive Primary Care initiative, *EHR* electronic health record, *HIE* health information exchange, *HIT* health information technology

## Results

In this section, we provide examples of actionable findings that emerged from our analysis. We define an actionable finding as one that provides information about changes that can be made to a program to improve its effectiveness or to program implementation to improve its uptake into practice. The actionable findings we present include descriptions of contextual factors important for understanding what happened in the primary care practices during CPC implementation and how those factors influenced the operationalization of CPC components into practice workflows.

We present example findings related to two CPC components (risk-stratified care management and care coordination) organized by the five CFIRs domains (Table 5). We include the CFIR construct from which the finding emerged in parentheses after each finding and then present how the finding informed actions to improve subsequent implementation of the intervention. One of the example findings we present in Table 5 links to the example analytic summaries that we present in Table 3, regarding respondents’ perceptions of the relative advantages of implementing care coordination. Across the 21 practices, from data segments coded to relative advantage and care coordination, we found that practice members perceived care coordination activities (e.g., contacting patients after a hospital discharge) to be beneficial because they ensured patient issues were not missed and moved work from the clinician to a nurse care manager who carried out important activities such as medication reconciliation. In Table 5, we present the action that was taken as a result of this finding; CMS and the learning-support providers communicated to practices the value of teamwork to take advantage of the skills of nurse care managers, reduce clinician burden, and ensure that important patient issues were not missed.

**Table 5:** Example actionable findings from selected CPC practices about implementing risk-stratified care management and care coordination and how the findings informed CPC implementation

CFIR domain	CPC component	Finding (CFIR construct)	Action
Intervention characteristics	Risk-stratified care management	Risk stratification and care management processes were seen as more complex and more time and resource intensive than anticipated. Practices faced challenges with documenting these activities and creating care plans in existing EHR systems. (Complexity)	CMS modified materials for practices about different approaches for carrying out risk stratification. The learning-support providers used this feedback to organize learning sessions and illustrative templates for practices about creating care plans with patients.
	Care coordination	Practice members perceived care coordination activities (e.g., contacting patients after a hospital discharge) as beneficial because they ensured patient issues did not slip through the cracks and moved work from the clinician to a nurse care manager who carried out important activities, such as medication reconciliation. (Relative advantage)	CMS and the learning-support providers provided practices with information about the value of teamwork to take advantage of the skills of nurse care managers, reduce clinician burden, and ensure important issues did not slip through the cracks.
Outer setting	Risk-stratified care management	Helping patients to self-manage chronic illness and make health-related lifestyle changes, particularly patients with limited social and economic resources, was identified by practice members as a common and time-consuming challenge to care management. (Patient needs and resources)	The extent of time and resources required to meet patients’ social needs and help them with economic barriers (e.g., need for transportation for an appointment) received more attention from CMS. For example, CMS emphasized such factors as part of risk stratification scores (patients with greater socioeconomic needs might be higher risk) in the following year’s implementation guidelines.
Inner setting	Risk-stratified care management	Practices had EHR systems in place, but those systems often lacked the functionality to support documentation related to risk-stratified care management. (Available resources)	CMS along with the learning-support providers created “affinity groups” to bring EHR vendor representatives and practices together to improve these EHR functions.
Characteristics of individuals	Risk-stratified care management	Practices that exhibited success in incorporating care management tended to have clinicians who believed in the value of care management and worked with patients and staff to incorporate the nurse care manager as part of the care team. (Knowledge and beliefs about the intervention)	Some health system-owned practices modified their care management workflows based on their first-year experiences to try to embed a care manager at the practice (rather than having him or her located at the corporate office).
Implementation process	CPC overall	One-on-one, tailored practice coaching and problem-focused learning (e.g., peer-to-peer learning on overcoming specific challenges) for individual practices was a key contributor to practice-level improvement efforts. (External change agents)	The learning-support providers increased opportunities for the practices to engage in peer-to-peer learning and (in certain cases) on-site practice coaching.

The findings presented in this table are from 2013. They are also presented in Ref [12]. *CFIR* Consolidated Framework for Implementation Research, *CMS* Centers for Medicare and Medicaid Services, *CPC* Comprehensive Primary Care, *EHR* electronic health record

The actions described in Table 5 were also informed by findings from the larger evaluation of the CPC initiative and by information CMS collected directly from practices, other payers, and other contractors. Overall, our findings and these other sources of feedback facilitated a collaborative approach to making program refinements over time.

## Discussion

In this article, we described our method for identifying and understanding contextual factors that influence the implementation of complex multicomponent health care delivery interventions.

Our approach to using the CFIR to develop interpretive codes to assess the influence of implementation context, in addition to using descriptive codes to delineate program components, guided us to generate actionable findings relevant to different primary care transformation activities. The codes we developed helped us to better understand how barriers and facilitators varied across the required components of the intervention and to discern which ones were common across all components. Together, this information produced findings that key evaluation stakeholders—CMS, the learning-support providers, and primary care practices—used to adapt intervention implementation guidelines and modify learning activities and supports to make them more relevant to practices.

Using the CFIR ensured that the key barriers and facilitators to implementation were examined systematically across CFIR domains and constructs, by prompting coders to critically interpret the meaning of each data segment when coding. By using the CFIR to organize the important contextual factors likely to influence the implementation of each CPC component, we were able to tell a comprehensive, organized, valid, and compelling story. Moreover, the story we told included actionable information that those supporting the implementation process could use to improve the success of the initiative in real time. Such timely, actionable findings during program implementation support a rapid-cycle approach to evaluation in which ongoing feedback is provided to program stakeholders to support learning, adaptation, and continuous quality improvement [6].

Our example findings demonstrate some of the factors that emerged as helpful or challenging for implementing two program components: risk-stratified care management and coordination of care across the medical neighborhood. We delineated the influence of different factors on different components to bring clarity to CPC stakeholders about how different practices are experiencing implementation of different aspects of a multicomponent intervention. For example, we described the nuances of challenges that some practices faced when lacking internal resources to document care management activities in the electronic health record (EHR), which prompted CMS and the learning-support providers to convene EHR vendors together with practices to facilitate problem solving. We described how teamwork enabled some practices to facilitate care transitions in the broader medical neighborhood, which helped learning-support providers promote the value of team-based care in improving care coordination. Decision makers can use such findings to design and improve future health care delivery interventions. Learning-support providers can use such findings to tailor support to implementing organizations, and implementing organizations can use such findings to avoid pitfalls and try approaches that were successful in other organizations.

The CFIR is a comprehensive typology of contextual factors that have been associated with effective implementation in published implementation theories and empirical studies. It provided a taxonomy or common language for our research team to identify, distill, and compare factors influencing primary care transformation across 21 practices, operating in different contexts. This approach is useful for project teams to divide coding and analysis, while maintaining a common orientation to the themes emerging from the data. However, we caution that the application of a large and multi-level codebook to complex data is an inherently difficult process that requires close attention to the quality and consistency of data collection, coding, and analysis.

We think the approach we describe has broad application and encourage others to use the CFIR, along with intervention-specific codes, to guide the efficient and rigorous analysis of rich qualitative data. As such, we are using this approach to assess barriers and facilitators to implementing a disease registry in the Supporting Practices to Adopt Registry-Based Care (SPARC) study [20]. The SPARC study is a two-armed randomized controlled trial of 30 primary care practices implementing a diabetes registry. Practices randomized to the intervention will receive learning support for registry implementation. Applying our approach to this study design will provide us the opportunity to demonstrate its relevance for assessing the implementation of a relatively smaller intervention and an implementation strategy designed to guide the implementation process.

As health care delivery interventions become more widespread, research studies and rigorous evaluations can be better compared when guided by the CFIR. The CFIR allows researchers across studies to use a common language and approach to comprehensively and systematically study implementation of multicomponent interventions. Researchers can better synthesize across settings, interventions, and studies their findings about factors that influence implementation to develop an evidence base for understanding implementation and developing theories to guide successful change [7, 21].

## Conclusion

This article demonstrates how we generated actionable findings that provided our evaluation client, CMS, learning-support providers, and primary care practices with information about the contexts underlying CPC implementation and how factors in those contexts may have influenced implementation progress. Findings derived using our systematic approach can inform stakeholders on how to change or improve implementation of an intervention in the current settings or replication of an intervention in different settings [22]. The CFIR can support the design of implementation studies by guiding analysis and reporting to generate findings that go beyond the documentation of intervention details and address important research questions about how, why, and under what conditions intervention implementation is effective. Our delineation of the multiple CPC program components, used in conjunction with CFIR constructs, guided our data collection, data analysis, and reporting and could be adapted to other studies evaluating the implementation of complex multicomponent interventions, within health care delivery and beyond.

## Additional files

Additional file 1: Excerpt from interview guide. (DOCX 32 kb)
Additional file 2: Excerpt from observation checklist. (DOCX 27 kb)
Additional file 3: Valence ratings. (DOCX 27 kb)

## Abbreviations

CFIR: Consolidated Framework for Implementation Research
CMS: Centers for Medicare and Medicaid Services
CPC: Comprehensive Primary Care
EHR: Electronic health record
