# Supplementary material for: Using the Consolidated Framework for Implementation Research (CFIR) to produce actionable findings: a rapid-cycle evaluation approach to improving implementation
Source: Implement Sci. 2017 Feb 10;12:15. doi: 10.1186/s13012-017-0550-7 (PMC5303301; doi:10.1186/s13012-017-0550-7)
Supplement: Additional file 1: — Excerpt from interview guide. (DOCX 32 kb) [file 13012_2017_550_MOESM1_ESM.docx]

**Experiences with the five CPC functional areas and Year 1 Milestones**

*For each CPC milestone/function, please probe on each question for information regarding:*

- *Changes to respondent’s role and training on new tasks/functions*
- *Others in the practice who are involved in the process*
- *Perceived challenges with the process*
- *Perceptions of what has been helpful with the process*
- *Perceptions of patient reactions*

1. What, if anything, is the practice doing to track and manage high-risk patients (**stratify patients by risk level)**?

[Follow-up]

1.a. How this changed in the past year?

*[If no changes identified]* Do you know if there are plans to make any changes related to tracking and managing high-risk patients? What are the plans?

2.b. What tools are being used to track and manage high risk patients? *[Ask for an example of how these tools are being used to manage the care of these patients.]* How have these tools changed in the past year?

1. What, if anything, is the practice doing about **improving how you coordinate patient care with other practices and organizations** in your area?

[Follow-up]

2.a. How has this changed in the past year?

*[If no changes identified]* Are you planning to make any changes to improve how you coordinate patient care with other practices in your area? What are the plans?

**CPC wrap-up**

1. Overall, what do you think of the CPC initiative?
2. What do you like the most about participating in this initiative?
3. What do you like the least about participating in this initiative?
4. What has helped your practice in its CPC work?
5. What challenges has your practice encountered in its CPC work?
