# Supplementary material for: Using the Consolidated Framework for Implementation Research (CFIR) to produce actionable findings: a rapid-cycle evaluation approach to improving implementation
Source: Implement Sci. 2017 Feb 10;12:15. doi: 10.1186/s13012-017-0550-7 (PMC5303301; doi:10.1186/s13012-017-0550-7)
Supplement: Additional file 2: — Excerpt from observation checklist. (DOCX 27 kb) [file 13012_2017_550_MOESM2_ESM.docx]

**Practice Descriptive Information**

1. Briefly describe the type of practice? (NOTE: Confirm existing information if already known)
   1. What implications does the practice’s structure have for daily work and the ability to chart its own course with respect to making changes in the practice?
2. Briefly describe the location of the practice (rural/urban, neighborhood characteristics, etc.)
   1. How well does the staff match the community in terms of cultural background/demographics/language?
3. Briefly describe the physical space and how this relates to staffing.
4. Describe the interactions between patients and staff members.
5. How well do practice members work together during the course of a patient visit?
6. How long have people been working here? How do staff members describe turnover?
7. How many clinicians are seeing patients at this site on a typical day? On the days of the observation?
8. How is the patient care team organized?
9. Does the practice have night or weekend hours? Who staffs the after-hours care? How do patients get access to these hours? What are they typically used for?
10. What mechanism does the practice have for building teams and facilitating communication and shared work processes between team members? (Does the practice have team meetings? If so, what are they for and how frequently do they occur? observe one if possible)

**Roles – collected by staff shadowing and informal interviewing**

Observe one practice member in each of the main roles in the practice (e.g., front desk, clinical support staff, clinician, nurse, office manager, biller, scheduler, care manager, etc.). Be sure to note if practice staff members are fulfilling more than one role.

1. What key functions are performed by each role?
   1. For each staff member who has time, ask/assess:
      1. What are your main responsibilities in the practice?
      2. Are you familiar with the Comprehensive Primary Care (CPC) Initiative? What is your practice doing for CPC? Can you tell me how CPC affected your responsibilities?
      3. Please show me how you … Specific activities we are interested in include: preparing the doctor for patient visits, rooming patients, scheduling patients, managing data for chronic illness care or prevention. How have these tasks changed since the start of the CPC initiative?
      4. Do you do anything specific for patients who are considered to be high risk? What do you do?
      5. If role involves making patient appointments (patient access), ask if patients are able to make appointments when needed (availability of appointment slots and their PCP).
      6. Do you reach out to patients to make appointments? How do you know what patients to reach out to?
      7. Do you assist patients in scheduling visits with other providers?
      8. How well do you know the patients that come to this practice? Why do you think you know them well, or don’t know them well?
