# Supplementary material for: Using the Consolidated Framework for Implementation Research (CFIR) to produce actionable findings: a rapid-cycle evaluation approach to improving implementation
Source: Implement Sci. 2017 Feb 10;12:15. doi: 10.1186/s13012-017-0550-7 (PMC5303301; doi:10.1186/s13012-017-0550-7)
Supplement: Additional file 3: — Valence ratings. (DOCX 27 kb) [file 13012_2017_550_MOESM3_ESM.docx]

Does construct reflect a FACILITATOR or BARRIER to the implementation of a CPC functional area?

| **FACILITATOR**  **POSITIVE Rating** | **BARRIER**  **NEGATIVE Rating** |
| --- | --- |
| If coded statement reflects the construct has a positive influence on the implementation of CPC (or CPC functional area).  1. Intervention Characteristics. Consider if the feature of CPC (or a CPC function) supports successful implementation.  2. Outer Setting. Consider if the feature of the environmental context supports successful implementation of CPC (or CPC functional area).  3. Inner Setting. Consider if the feature of the practice (or in some cases the system with which the practice is affiliated) supports successful implementation of CPC (or CPC functional area).  4. Characteristics of Individuals. Consider if the characteristics of individuals support successful implementation of CPC (or CPC functional area).  5. Process. Consider if what occurred within the practice to implement CPC supports successful implementation of CPC (or CPC functional area). | If coded statement reflects the construct has a negative influence on the implementation of CPC (or CPC functional area).  1. Intervention Characteristics. Consider if the feature of CPC (or a CPC function) hinders successful implementation.  2. Outer Setting. Consider if the feature of the environmental context hinders successful implementation of CPC (or CPC functional area).  3. Inner Setting. Consider if the feature of the practice (or in some cases the system with which the practice is affiliated) hinders successful implementation of CPC (or CPC functional area).  4. Characteristics of Individuals. Consider if the characteristics of individuals hinder successful implementation of CPC (or CPC functional area).  5. Process. Consider if what occurred within the practice to implement CPC hinders successful implementation of CPC (or CPC functional area). |

**NEUTRAL Rating**: A coded statement does not reflect the construct to have a positive or negative influence on the implementation of CPC or outcomes. A neutral rating may also result from respondents contradicting each other (with credible information) or the construct may manifest both positively and negatively at different levels.
